# Recognition of Poly(A) RNA through Its Intrinsic Helical Structure

**DOI:** 10.1101/sqb.2019.84.039818

**Published:** 2020-04-15

**Authors:** Terence T.L. Tang, Lori A. Passmore

**Affiliations:** MRC Laboratory of Molecular Biology, Cambridge CB2 0QH, United Kingdom

## Abstract

The polyadenosine (poly(A)) tail, which is found on the 3’ end of almost all eukaryotic messenger RNAs (mRNAs), plays an important role in the posttranscriptional regulation of gene expression. Shortening of the poly(A) tail, a process known as deadenylation, is thought to be the first and rate-limiting step of mRNA turnover. Deadenylation is performed by the Pan2–Pan3 and Ccr4–Not complexes that contain highly conserved exonuclease enzymes Pan2, and Ccr4 and Caf1, respectively. These complexes have been extensively studied, but the mechanisms of how the deadenylase enzymes recognize the poly(A) tail were poorly understood until recently. Here, we summarize recent work from our laboratory demonstrating that the highly conserved Pan2 exonuclease recognizes the poly(A) tail, not through adenine-specific functional groups, but through the conformation of poly(A) RNA. Our biochemical, biophysical, and structural investigations suggest that poly(A) forms an intrinsic base-stacked, single-stranded helical conformation that is recognized by Pan2, and that disruption of this structure inhibits both Pan2 and Caf1. This intrinsic structure has been shown to be important in poly(A) recognition in other biological processes, further underlining the importance of the unique conformation of poly(A).

Almost all mature eukaryotic messenger RNAs (mRNAs) carry a 3’ polyadenosine (poly(A)) tail. As a nascent mRNA emerges from RNA polymerase II (Pol II), it is specifically recognized, cleaved, and polyadenylated by the cleavage and polyadenylation factor (CPF in yeast, CPSF in mammals) (Proudfoot 2011). The length of the mature poly(A) tail ranges from ~70 nt in yeast (Brown and Sachs 1998) to ~250 nt in mammalian cells (Brawerman 1981). The poly(A) tail is required for the export of mature mRNA into the cytoplasm (Fuke and Ohno 2008), where it is bound by the cytoplasmic poly(A)-binding protein (Pab1 in yeast, PABPC1 in mammals) (Kuhn and Wahle 2004). In the cytoplasm, poly(A) tails are shortened: In general, the poly(A) tail length of cytoplasmic mRNAs is much shorter than that synthesized in the nucleus (Chang et al. 2014; Lima et al. 2017; Eisen et al. 2020).

The poly(A) tail regulates posttranscriptional gene expression through multiple mechanisms (Fig. 1). First, the poly(A) tail increases the efficiency of translation initiation. This is thought to occur through protein–protein interactions between the cytoplasmic poly(A)-binding protein on the poly(A) tail and the eIF4G subunit of the cap-binding complex at the 5’ cap (Sachs 1990; Tarun and Sachs 1996; Hentze 1997), effectively circularizing the transcript. A circularized form of mRNA has been directly observed by atomic force microscopy in vitro (Wells et al. 1998), but controversy remains regarding whether or not the circularized form of mRNA is prevalent in cells (Pierron and Weil 2018; Vicens et al. 2018).

Second, the poly(A) tail is important for mRNA stability; shortening of the poly(A) tail, or deadenylation, is the first and rate-limiting step of mRNA decay for most eukaryotic transcripts (Chen and Shyu 2011). Once the poly(A) tail is removed, decapping and further 3’–5’ or 5’–3’ degradation can occur. Thus, deadenylation is an important step in the posttranscriptional regulation of gene expression because it determines transcript half-life (Wilson and Treisman 1988; Meyer et al. 2004; Parker and Song 2004). Indeed, deadenylation has been implicated in physiological processes such as development (Nakamura et al. 2004; Morita et al. 2007; Neely et al. 2010) and tumorigenesis (Faraji et al. 2016). In eukaryotes, deadenylation is primarily performed by two highly conserved multiprotein complexes, Pan2–Pan3 and Ccr4–Not, that shorten the poly(A) tail in a 3’–5’ direction. Within these complexes, the exonucleases that carry out deadenylation are Pan2/PAN2, and Ccr4/CNOT6/CNOT6L and Caf1/ CNOT7, respectively.

Deadenylase complexes are thought to degrade only the poly(A) tail and not the transcript body. This specificity is thought to arise through the intrinsic specificity of the exonuclease enzymes for adenosines, as well as through specific interactions of the poly(A) tail with subunits of the conserved deadenylase complexes. Here, we review the molecular basis of poly(A) specificity in deadenylation. These data reveal the importance of the unique intrinsic conformation of the poly(A) tail that is also exploited for its recognition in several other biological processes.

## Poly(A) Recognition by Deadenylase Complexes

**Figure 2:**
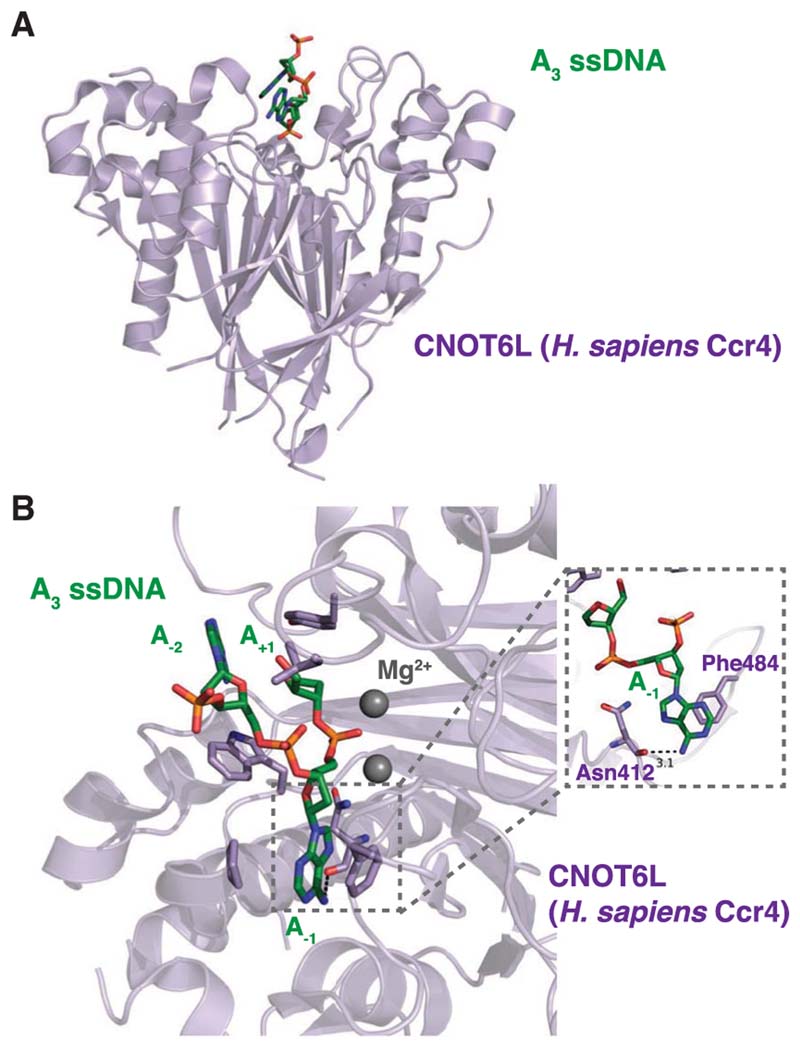
Recognition of poly(A) by *Homo sapiens* CNOT6L. A crystal structure of the Ccr4 deadenylase bound to poly(A) DNA (PDB: 3NGO) revealed the molecular determinants of poly(A) specificity of CNOT6L (Ccr4 in other organisms). (*A*) An overview of the CNOT6L structure (purple, shown as cartoon) bound to poly(A) DNA (green, shown as sticks). (*B*) A close-up view of poly(A) DNA in the CNOT6L active site. The scissile phosphate group is coordinated by two metal ions (shown as spheres) in the active site, which in turn is lined by hydrophobic residues (shown as sticks). (*Inset*) Interactions of the adenosine 5’ to the scissile phosphate (A_-1_) with the carbonyl oxygen of Asn412 and the phenyl ring of Phe484.

The activities of the *Schizosaccharomyces* *pombe* and Homo sapiens Ccr4–Not complexes have been shown to be specific for adenosines in in vitro deadenylation assays as the complexes stall upon encountering non-A stretches upstream of the poly(A) tail (Stowell et al. 2016; Raisch et al. 2019). The molecular basis of poly(A) recognition by Ccr4 has been elucidated through a crystal structure of human CNOT6L bound to single-stranded poly(A) DNA (Fig. 2A; Wang et al. 2010). In this structure, poly(A) DNA is bound in the active site cleft of the heart-shaped CNOT6L enzyme and the scissile phosphate group points into the base of the cleft toward the active site residues. The interactions between Ccr4 and poly(A) suggest that the specificity for adenine is determined by a hydrogen bond between the carboxyl oxygen of Asn412 and the N6 amine group of the penultimate adenine (A_-1_), as well as a stacking interaction between the aromatic adenine base and Phe484 (Fig. 2B). Nonetheless, this study did not address whether or not other subunits of the Ccr4–Not complex contribute to the recognition of the poly(A) tail, and how Caf1, the other exonuclease of the Ccr4–Not complex, specifies for poly(A).

Within the other major deadenylase complex, Pan2–Pan3, several domains and motifs contribute to poly(A) specificity. Pan2–Pan3 is composed of one Pan2 and two Pan3 molecules (Jonas et al. 2014; Schäfer et al. 2014, 2019; Wolf et al. 2014). The Pan3 subunit binds to the carboxy-terminal domain of cytoplasmic poly(A)-binding protein via a polypeptide stretch known as the PABP-interacting motif 2 (PAM2 motif), thereby recruiting the Pan2–Pan3 complex to the poly(A) tail (Siddiqui et al. 2007). Moreover, Pan3 contains an amino-terminal zinc finger domain that specifically binds poly(A) (Wolf et al. 2014). Thus, Pan3 contributes to the recognition of poly(A) RNA, but it was unclear whether the exonuclease domain of Pan2 also contained intrinsic specificity for adenosine.

## Specificity of Pan2 and Caf1 Exonucleases

**Figure 3:**
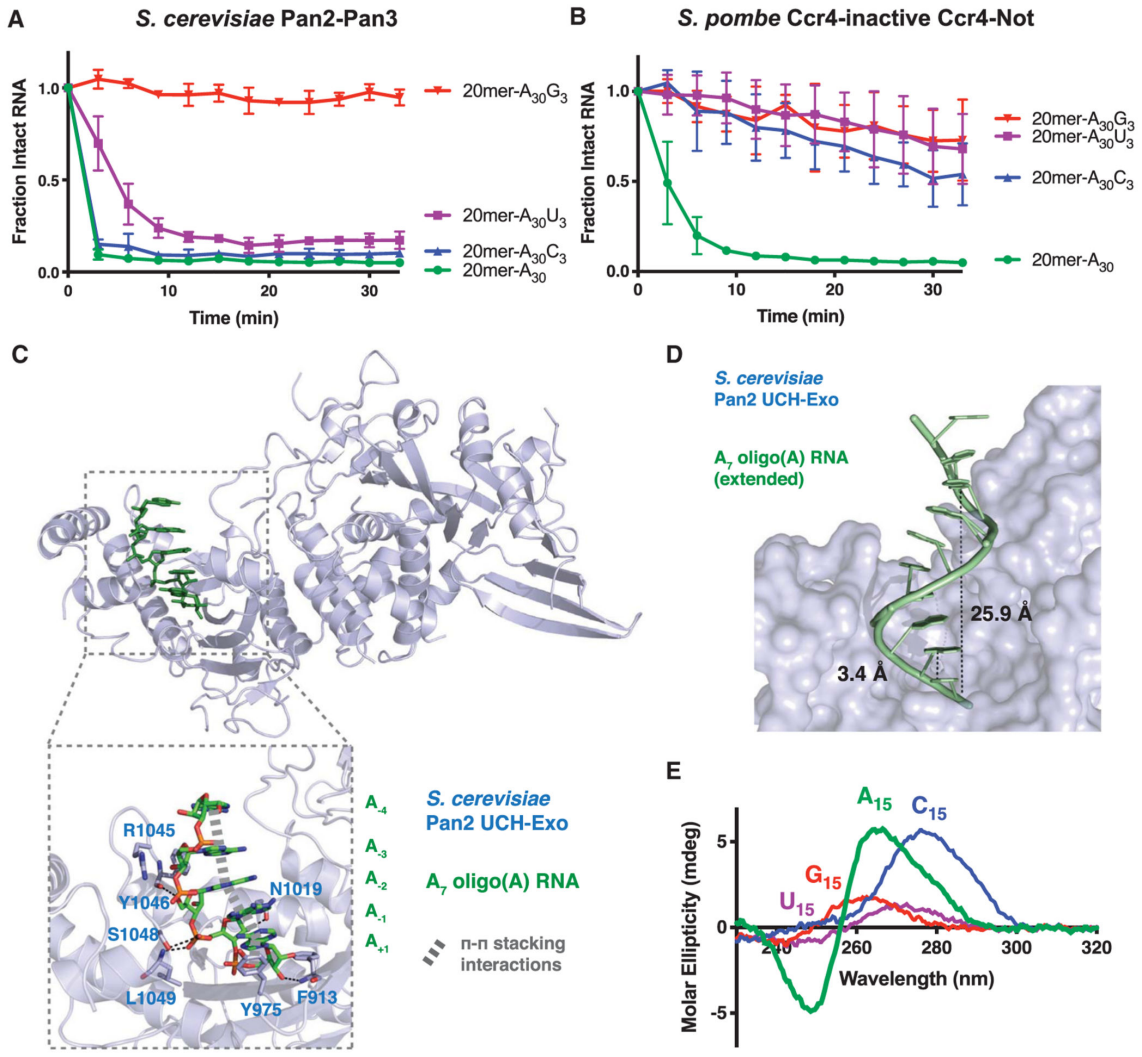
Nucleotide specificity of DEDD deadenylases. (A,B) In vitro deadenylation by *Saccharomyces cerevisiae* Pan2–Pan3 (*A*) or *Schizosaccharomyces pombe* Ccr4-inactive Ccr4–Not (*B*) on RNA substrates with a 30-nt poly(A) tail harboring different 3’ ribonucleotides. The disappearance of the substrate was quantified by intensity measurements of the band corresponding to the intact, fluorescently labeled RNA. Data were normalized to that at time = 0 and individual points are connected by straight lines for clarity. Assays were performed in triplicate; the points represent the mean, and the error bars represent standard deviation. (Reproduced from Tang et al. 2019, Figs. 1B and 2C.) (*C*) Crystal structure of the Pan2 UCH and exonuclease (Exo) domains (blue, shown as cartoon) bound to a poly(A) RNA (green, shown as sticks) (PDB: 6R9J). (*Inset*) Protein–RNA interactions in the Pan2 active site. Amino acids involved in the interaction are shown as sticks and labeled in blue. Putative hydrogen bonds are indicated with black dashed lines, and π–π stacking interactions are indicated with thick gray dashes. (*D*) Extended oligo(A) helix bound to Pan2, modeled by duplication and superposition of the observed A_5_. Distances are shown in Angstroms. (*E*) Circular dichroism spectra of A_15_ (green), U_15_ (purple), C_15_(blue), and G_15_ (red) RNAs. (Reproduced from Tang et al. 2019, Fig 7B.)

To determine the nucleotide specificities of the DEDD exonucleases Caf1 and Pan2, we used in vitro biochemical assays with recombinant Pan2–Pan3 or with Ccr4–Not containing a catalytic mutant of Ccr4 (such that Caf1 was the only active nuclease) (Tang et al. 2019). Both enzymes showed a preference for poly(A) when incubated with fluorescently labeled RNA substrates containing poly(A) tails with varied 3’-terminal nucleotides (A_30_-U_3_,-C_3_,-G_3_) (Fig. 3A,B). Caf1 shows strict specificity for poly(A) and is inhibited by all non-A nucleotides, whereas Pan2 is substantially inhibited only by guanosines at the end of a poly(A) tail (Fig. 3A,B). These specificities likely prevent the 3’ untranslated region (UTR) of the transcript from being degraded by deadenylase complexes. Notably, both deadenylase complexes are generally inhibited when they reach the end of the poly(A) tail.

## Non-a Sequences in Poly(A) Tails

In cells, the poly(A) tail can be modified by the addition of other nucleotides. After deadenylation, shortened oligo(A) tails can be marked by an oligo(U) tail deposited by terminal uridyl transferases (TUTases) to label specific transcripts for degradation (Rissland and Norbury 2009; Lim et al. 2014). Recent studies have also identified that non-A nucleotides can be incorporated throughout mammalian poly(A) tails at a low frequency (Chang et al. 2014; Legnini et al. 2019) by the noncanonical poly(A) polymerases TENT4A (PAPD7) and TENT4B (PAPD5) (Lim et al. 2018). The presence of guanosines within the poly(A) tail correlates with increased transcript half-life, suggesting that these modifications may affect transcript stability (Chang et al. 2014).

The lack of inhibitory effect by uracils on Pan2–Pan3 and Ccr4–Not suggests that the oligo(U) tail alone does not impede deadenylation (Tang et al. 2019). Nonetheless, an oligo(U) tail is thought to recruit RNA-binding proteins, such as the Lsm complex, that could block deadenylation (Song and Kiledjian 2007). In contrast, the presence of guanosines within the poly(A) tail inhibits both Pan2–Pan3 and Ccr4–Not (Fig. 3A,B). This in vitro result agrees with the observation that guanylated poly(A) tails correlate positively with transcript half-life, suggesting that guanylation could be a mechanism by which transcripts are selectively stabilized by inhibition of deadenylation. It remains unclear whether the incorporation of non-A nucleotides into the poly(A) tail is a regulated or stochastic process, and how significant this process is in regulating gene expression in a global or transcript-specific manner.

## Poly(A) Recognition by Pan2

We used X-ray crystallography to further investigate DEDD deadenylase specificity. Previous crystal structures of the carboxy-terminal half of Pan2 in the absence of RNA had revealed that it consists of a pseudo–ubiquitin hydrolase (UCH) domain and an exonuclease (Exo) domain with the two domains forming a contiguous structural unit (UCH-Exo) (Jonas et al. 2014; Schäfer et al. 2014). We determined the molecular basis of poly(A) recognition by the Pan2 exonuclease from a crystal structure of a UCH-Exo catalytic mutant from *Saccharomyces cerevisiae* bound to oligo(A) RNA (Fig. 3C; Tang et al. 2019). Pan2 does not undergo any major conformational changes upon substrate binding, suggesting that the UCH-Exo domains are rigid. Although one of the metalcoordinating residues was mutated to prevent RNA degradation, the RNA scissile phosphate bond faced the key catalytic residues within the active site, consistent with productive RNA binding. Furthermore, this structure revealed the contacts between Pan2 and oligo(A) RNA in the active site, including a π-stacking interaction between the terminal adenine and the phenyl group of Y975, as well as putative hydrogen bonds between amino acid residues (F913, N1019, Y1046, S1048, and L1049) and the ribophosphate backbone (Fig. 3C, inset). Surprisingly, apart from the stacking interaction of the terminal adenine, there were no interactions between Pan2 and the adenine bases, raising the question of how Pan2 specifically recognizes poly(A).

## The Intrinsic Structure of Poly(A)

Within the crystal structure, oligo(A) formed a singlestranded A-form-like helix in the Pan2 active site, where each adenine base was π-stacked in an offset parallel manner onto adjacent bases (Fig. 3C, inset). This suggested that Pan2 may recognize the shape of the RNA, instead of directly binding functional groups specific to adenine.

The in vitro conformation of poly(A) RNA has been extensively investigated by circular dichroism (CD) (Brahms et al. 1966; Hashizume and Imahori 1967), temperature jump studies (Dewey and Turner 1979), and crystallography (Suck et al. 1976). More recently, the conformation has been further studied by atomic force microscopy (Smith et al. 1997), optical tweezers (Seol et al. 2007), nuclear magnetic resonance (NMR) (Isaksson et al. 2004), and protein nanopores (Lin et al. 2010). From these studies, it had been proposed that poly(A) can form a single-stranded A-form helix at physiological pH in solution, with the adenine bases stacked in a roughly parallel orientation (Saenger 1984; Bloomfield et al. 1999). The helical conformation adopted by oligo(A) in the Pan2 active site (Fig. 3D) is similar to that hypothesized by Saenger et al. (1975) derived from the stacked configuration of two adenosines in a crystal structure of A_3_ RNA. Given the lack of base-specific contacts between adenine functional groups and the protein, our data suggested that Pan2 recognizes the intrinsic structure formed by oligo(A).

To assess if poly(A) RNA forms an intrinsic structure in solution in the absence of protein, we used CD to study 15-mer polyribonucleotides. CD spectra are sensitive to higher-order chiral structures formed by a macromolecule. Poly(A) is unique in forming a signature peak (265-nm) and trough (250-nm) structure, which cannot be found with other polyribonucleotides (Fig. 3E). Interestingly, poly(C) adopts a different structure with a peak at 278 nm, presumably corresponding to a previously solved crystal structure of poly(C) RNA (Akinrimisi et al. 1963; Arnott et al. 1976) with different characteristic helical parameters relative to poly(A). Thus, in vitro, poly(A) RNA forms a unique structure in solution compared to other polyribonucleotides.

## The Stacked, Helical Structure of Poly(A) is Important in Deadenylation

**Figure 4:**
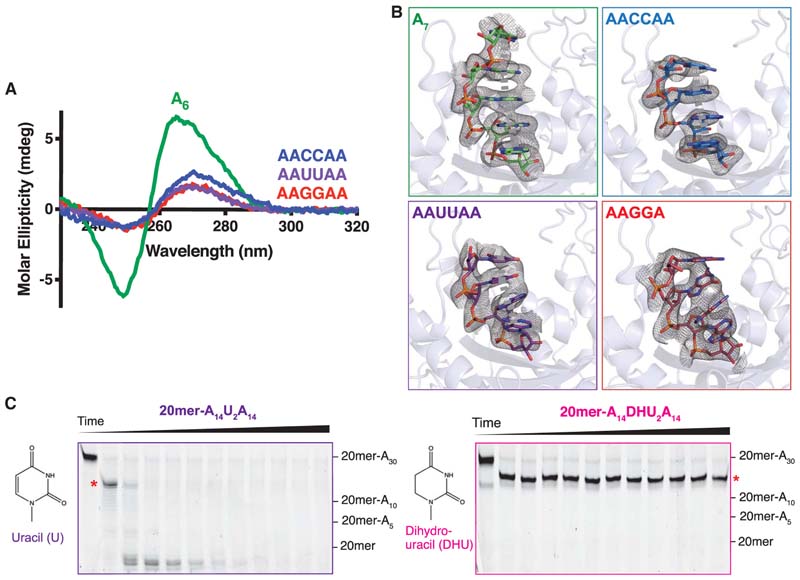
A stacked helical conformation of poly(A) RNA is required for Pan2 activity. (*A*) Circular dichroism spectra of A_6_ (green), AAUUAA (purple), AACCAA (blue), and AAGGAA (red) RNAs. (*B*) Crystal structures of Pan2 UCH-Exo (shown as light blue cartoon) bound to oligo(A) RNAs (shown as green sticks) interrupted by two uracils (purple, PDB: 6R9P), cytosines (blue, PDB: 6R9Q), or guanosines (red, PDB: 6R9O). The electron density shown represents feature-enhanced maps (FEM) contoured to 1.8 σ. π-stacking interactions between adjacent bases, if any, are denoted in thick dashed lines in gray. (*C*) In vitro deadenylation assays of Pan2 UCH-Exo on fluorescently labeled RNAs with 30-nt poly(A) tails interrupted either by two uracils (*left*, purple) or two dihydrouracils (*right*, pink). The chemical structures of uracil and dihydrouracil are shown alongside each assay for reference. The red asterisk indicates the poly(A) tail length at which stalling occurs (-UUAAA or -DDAAAA). (Reproduced from Tang et al. 2019, Fig, 6C,D.)

In vitro deadenylation assays showed that Pan2 was not strongly inhibited by uracils (Us) or cytosines (Cs) at the end of a poly(A) tail (Fig. 3A,B), but these nucleotides did not show the characteristic CD signature of helical poly(A), either alone (Fig. 3E) or in the context of oligo (A) (Fig. 4A). If Pan2 recognizes the unique helical structure of poly(A) in its active site, how does it remove these non-A nucleotides? In crystal structures of Pan2 bound to different oligonucleotides, we observed that oligo(A) RNA containing two Us or Cs forms a similar stacked, helical structure to poly(A) in the active site (Fig. 4B; Tang et al. 2019). Thus, C- and U-containing RNAs can form the π-stacking interactions necessary for the helical conformation while bound to the Pan2 active site, further suggesting that Pan2 specifically recognizes the formation of a poly(A) helix-like structure. As these C- and U-containing RNAs do not adopt an intrinsic poly(A)-like structure, the formation of this structure in the Pan2 active site likely comes at an entropic cost, leading to a small reduction in Pan2 activity on these substrates.

In contrast, the presence of guanosines (Gs) disrupts the stacked, helical structure of poly(A) as the crystal structure revealed that the G-containing RNA is unstacked in the Pan2 active site (Fig. 4B; Tang et al. 2019). This unstacking likely disrupts the correct recognition of the ribophosphate backbone and in particular the scissile phosphate bond, leading to the inhibition of deadenylation. Although previous studies of dinucleotides have predicted that guanosines have energetically favorable stacking interactions with each other and with adenosines (Friedman and Honig 1995; Brown et al. 2015), the configurations of these stacked guanosine dinucleotides cannot be accommodated within an A-form-like single-stranded RNA helix. This leads to unstacking within the context of poly(A) and disruption of ideal helical geometry.

Pan2 recognition of the stacked, helical form of poly(A) was further tested using modified nucleotides that inhibit stacking. Dihydrouracil (DHU) is a uracil analog that contains the same functional groups as uracil, except for a C–C single bond between C5 and C6 instead of a C=C double bond. As such, DHU is nonplanar and disrupts stacking interactions between adjacent bases. The introduction of two DHUs into a poly(A) tail strongly inhibits Pan2 activity relative to two uracils, which only cause a slight stall in deadenylation (Fig. 4C; Tang et al. 2019). This supports the finding that disruption of base-stacking inhibits Pan2, and that Pan2 requires its substrate to adopt a poly(A)-like stacked, helical conformation.

## Effect of Adenine Modifications on Deadenylation

**Figure 5:**
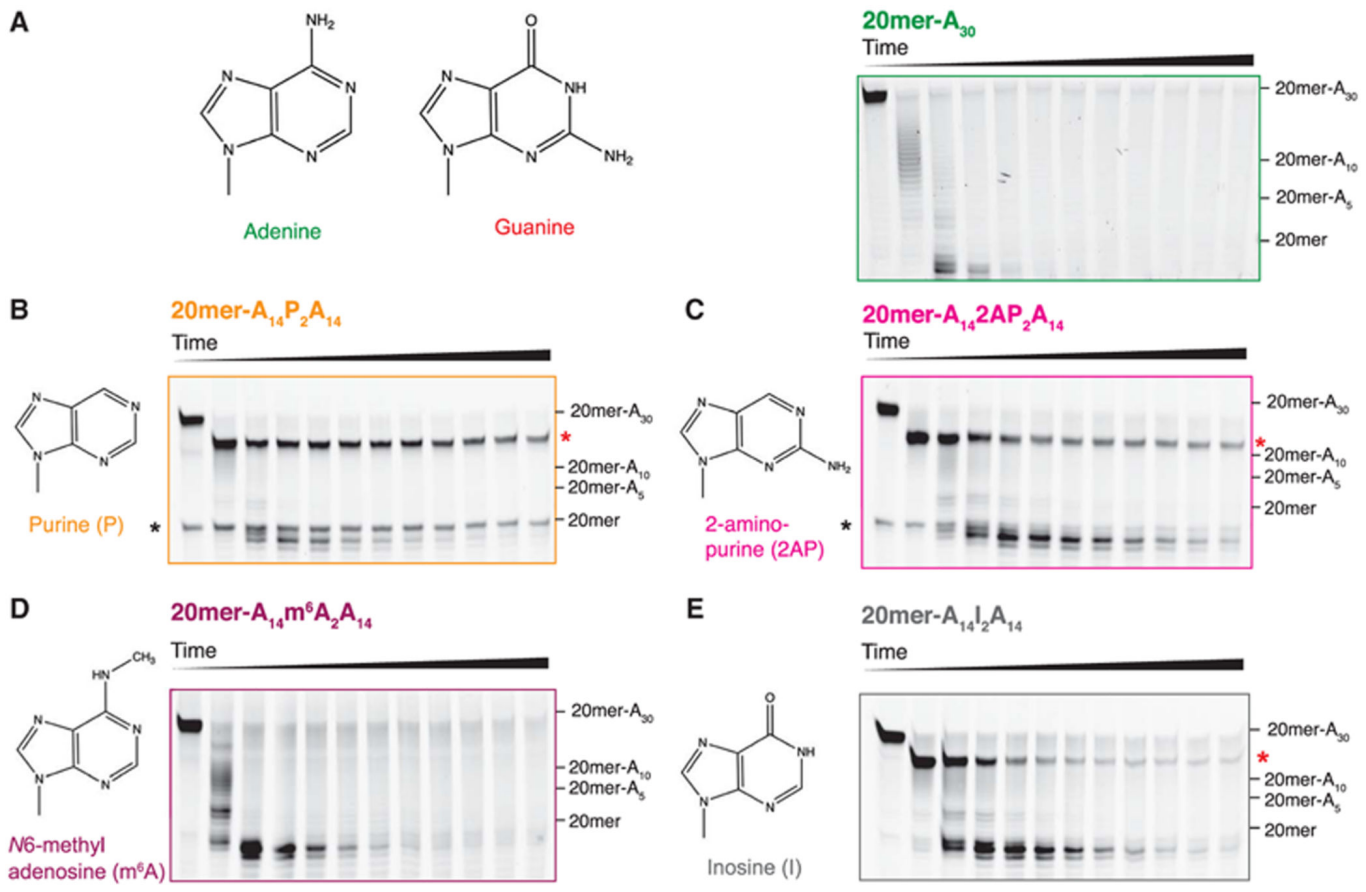
Effect of modified nucleotides on Pan2 activity. In vitro deadenylation assays of Pan2 UCH-Exo on fluorescently labeled RNAs with 30-nt poly(A) tails (*A*) or 30-nt poly(A) tails interrupted by two purine (*B*, orange), two 2-aminopurine (*C*, magenta), two N6-methyladenosine (*D*, dark purple), or two inosine (*E*, gray) nucleotides. The chemical structures of the corresponding bases are shown beside each assay for reference. The red asterisk indicates the poly(A) tail length at which stalling occurs. The black asterisk indicates a contaminating nucleic acid band.

Why does the planar guanine disrupt stacking of a poly(A) helix? Guanines and adenines differ in the position of an amino group and the addition of a carbonyl group around the purine moiety (Fig. 5A). This affects the electronic distribution within the aromatic ring system through inductive and resonance effects. The distribution of functional groups in adenine likely enables electrostatic complementarity when adenines are stacked on top of each other in a single-stranded helix. To test this hypothesis, we designed RNA substrates with poly(A) tails interrupted by two purines (Ps), which lack the amine group on C6 (Fig. 5B), as well as two 2-aminopurines (2APs), which lack the amine group on C6 and contain an additional amine group on C2, compared to adenine (Fig. 5C). These were then tested in deadenylation assays with Pan2 and compared to an RNA containing a pure poly(A) tail (Fig. 5A; TTL Lang, LA Passmore, unpubl. data). The introduction of purines or 2APs results in a strong inhibition of Pan2 exonuclease activity (Fig. 5B,C; TTL Lang, LA Passmore, unpubl.). This is in agreement with an important role for the electrostatic distribution of functional groups in forming the stacked, helical conformation of poly(A). If the functional groups within adenine are removed or altered in position, as in the case of purines, 2APs, and guanines, the poly(A)-like structure is disrupted, which in turn inhibits Pan2. Thus, the electronic distribution around the central purine of adenine is unique and likely enables its intrinsic stacked, helical structure.

Adenine can be chemically modified in vivo by the addition of a methyl group onto the N6 amine to form N^6^-methyladenosine (m^6^A) (Shi et al. 2019). This modification can be specifically recognized by diverse RNA-binding proteins, regulating processes such as RNA degradation (Du et al. 2016) and splicing (Xiao et al. 2016). Adenine can also be deaminated at the N6 position to inosine (Alseth et al. 2014), which has been reported in mRNAs (Paul and Bass 1998). Deamination of adenine has been implicated in numerous human diseases such as psychiatric disorders and cancers (Slotkin and Nishikura 2013). Importantly, these modifications can cause subtle changes in the electronic distribution of adenine.

To test whether these modifications affect the stacked, helical conformation of poly(A) and thus deadenylation by Pan2, we introduced two m^6^As or two inosines into the poly(A) tail and tested the substrates in deadenylation assays with Pan2 (Fig. 5D,E: TTL Lang, LA Passmore, unpubl. data). We observed that m^6^A had almost no effect on deadenylation by Pan2 relative to the unmodified poly (A) tail (Fig. 5D). In contrast, when Pan2 encounters inosines, there is a stall in deadenylation (Fig. 5E).

To date, m^6^A and inosine nucleotides have not been identified in the poly(A) tail; however, limitations in sequencing techniques would have precluded their detection. We expect that the increased sensitivity and improvements in sequencing techniques, such as long-read nanopore sequencing, will be able to detect such modifications if they occur within the poly(A) tail (Liu et al. 2019). Overall, our results show that the formation of the helical conformation is dependent on the electronic distribution in adenine, enabling electrostatic complementarity upon adenine base-stacking.

## The Poly(A) Structure in other Biological Processes

**Figure 6:**
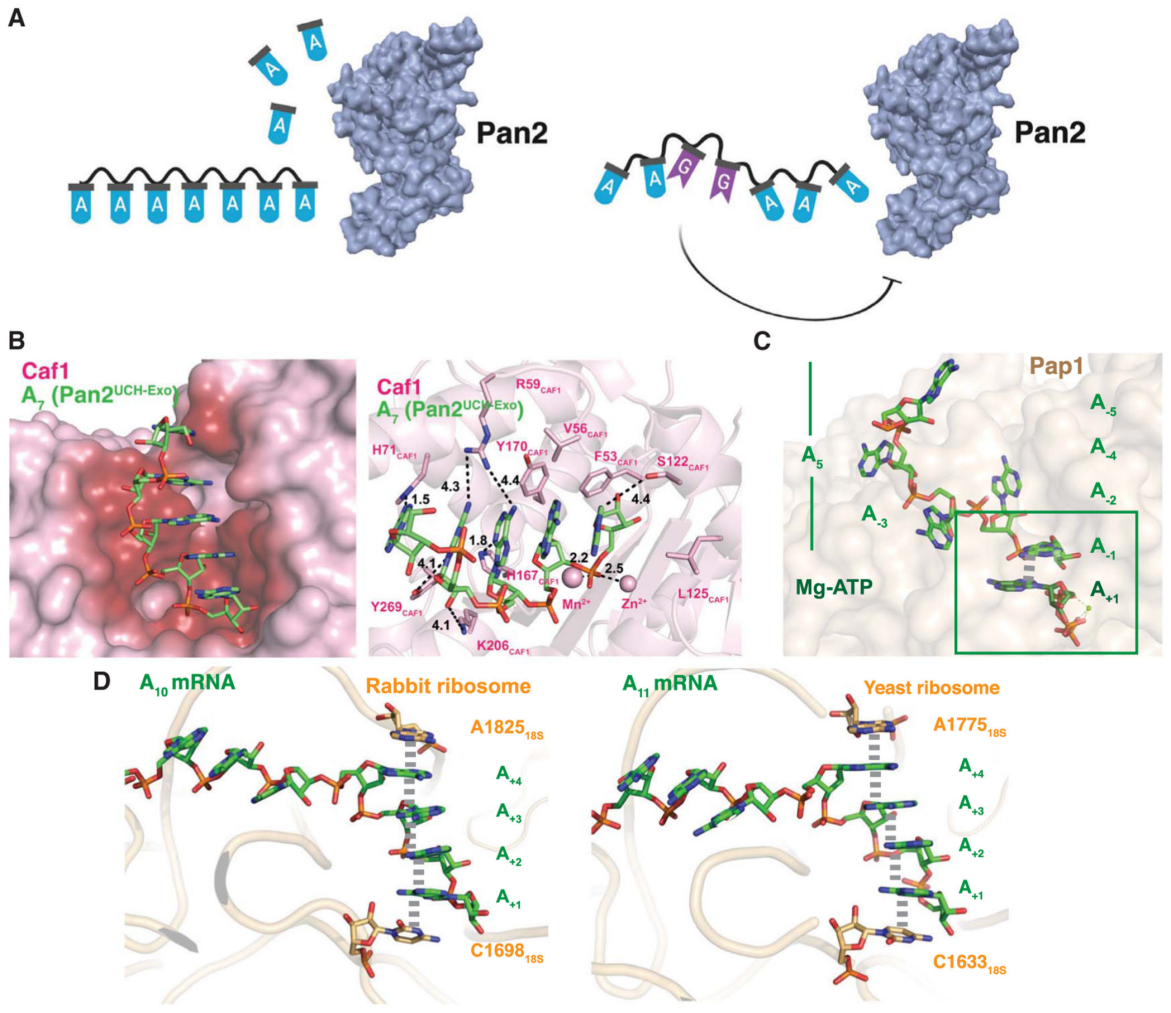
Role of the stacked, single-stranded poly(A) helix in biological processes. (*A*) Proposed model for the recognition of poly(A) RNA by Pan2 through its stacked, single-stranded, helical conformation. Disruption of this conformation inhibits Pan2. (*B*) Proposed model of recognition of poly(A) (shown as green sticks) by Caf1 (shown as pink surface and as cartoon; PDB: 3G0Z). The Caf1 active site (*left*, surface) is colored by proximity to the poly(A) substrate, showing that poly(A) is more buried compared to in the Pan2 active site. Putative protein-RNA contacts between amino acid side chains (labeled, shown as sticks) and the poly(A) substrate are shown in the *right* panel. Possible hydrogen bonds are shown as black dashed lines and are labeled with distance, shown in Ångstroms. (Reproduced from Tang et al. 2019, Supplemental Figs. 4A,B.) (*C*) Structure of yeast poly(A) polymerase Pap1 (shown as khaki surface) bound to poly(A) RNA and an incoming ATP (shown as green sticks) (PDB: 2Q66). The 3’-terminal adenine appears to stack against the adenine of the incoming ATP; the π-stacking interaction is depicted with a thick gray dash. (*D*) Structures of oligo(A) RNA (shown in sticks, green) bound to rabbit ribosome (*left*, shown as orange cartoon; PDB: 6SGC) or yeast ribosome (*right*, shown as orange cartoon; PDB: 6T7T). The oligo(A) RNA is stacked between an adenine and cytosine (shown as orange sticks) of the 18SrRNA. π-stacking interactions are shown as thick gray dashes.

Although a structure of poly(A) had been previously proposed, it had never been directly observed, and the biological significance of the helical conformation was unclear. Together, our data show that the intrinsic single-stranded helix of poly(A) is exploited by Pan2 for specificity and recognition, and that disruption of this structure is sufficient to inhibit Pan2 (Fig. 6A).

Caf1 is a DEDD-family exonuclease in the Ccr4–Not complex with structural homology with the Pan2 exonuclease domain. Thus, we hypothesized that Caf1 would also recognize the stacked, helical structure of poly(A). We were unable to obtain crystals of the DEDD deadenylase Caf1 in complex with oligo(A) RNA, but we could model the poly(A) helix into the active site of a previously determined structure of *S*. *pombe* Caf1 (Andersen et al. 2009). This showed that the poly(A) helix can be accommodated in the Caf1 active site, forming plausible contacts between the ribophosphate backbone and side chains of Caf1 (Fig. 6B). Similar to Pan2, Caf1 is strongly inhibited by DHU relative to uracils, suggesting that it may also recognize the stacked, helical conformation of poly(A) (Tang et al. 2019). However, our modeling shows that there are also putative base-specific contacts, consistent with our observation that Caf1 shows greater nucleotide specificity compared to Pan2 (Tang et al. 2019).

Adenine stacking has also been observed in the context of polyadenylation, the process whereby adenosines are processively added by a poly(A) polymerase to the 3’ end of a nascent transcript (Kumar et al. 2019). A structure of yeast poly(A) polymerase in complex with ATP and oligo (A) has been determined, providing a model for how adenosines are added to the 3’ end of an elongating poly(A) tail (Balbo and Bohm 2007). Most adenines are flipped out to form base-specific contacts with the Pap1 active site (A_-2_, A_-3_, A_-4_, A_-5_) but, interestingly, the incoming ATP appears to π-stack against the terminal adenine of the existing poly(A) tail (A_-1_). This mode of stacking is reminiscent of the π-stacking observed in the Pan2 active site, suggesting that the stacking geometry of an incoming ATP against the existing 3’ adenosine contributes to the specificity of adenosine addition by Pap1.

Two recent studies have revealed a role for the singlestranded poly(A) helix during translation of a poly(A) tail by the ribosome (Chandrasekaran et al. 2019; Tesina et al. 2020). Normally, translation is terminated at a stop codon before the ribosome reaches the poly(A) tail. If the ribosome encounters a poly(A) stretch, the ribosome stalls and a quality control pathway results in degradation of the transcript and disassembly of the stalled ribosome (Doma and Parker 2006; Shao et al. 2013; Juszkiewicz and Hegde 2017; Sundaramoorthy et al. 2017). In both rabbit and yeast ribosomes, poly(A) RNA forms a singlestranded RNA helix in the A-site of the ribosome, stacking between 18S rRNA bases A1825 and C1698, leading to a structural rearrangement in the decoding center of the ribosome (Fig. 6D). This contributes to ribosome stalling. Analogous to the recognition of the poly(A) helix by Pan2, disruption of the poly(A) helix in the decoding center by the introduction of guanosines—for instance, when poly(A) is replaced with an (AAG)_n_ tract—leads to disruption of the poly(A) helix, and the ribosome thus does not stall (Arthur et al. 2015; Juszkiewicz and Hegde 2017; Sundaramoorthy et al. 2017). The ability of poly(A) to form a single-stranded helix is thereby recognized and exploited in translational quality control.

## Conclusion

The recent work described here shows that proteins can recognize the sequence of RNA, particularly poly(A), through the intrinsic conformation of single-stranded RNA. This is reminiscent of the mechanism whereby DNA binding proteins (such as the Trp repressor) use indirect readout to recognize specific DNA sequences (Otwinowski et al. 1988). Poly(A) RNA likely interconverts between helical and unstructured conformations in solution. In the Pan2 active site, this helical conformation is stabilized by contacts with the ribophosphate backbone. Interestingly, the characteristic CD signature of adenosine stacking can be observed with oligo(A) polyribonucleotides as short as A3 (TTL Lang, LA Passmore, unpubl. data). Proteins can also recognize the poly(A) tail via base-specific interactions. For instance, in the poly(A) polymerase active site, the terminal adenosines within the existing poly(A) tail contact the protein via specific functional groups (Balbo and Bohm 2007). Similarly, the RNA recognition motif domains (RRMs) of the cytoplasmic poly(A) binding protein recognize poly(A) RNA through interactions with base-specific groups with the RNA in an extended, nonhelical conformation (Deo et al. 1999; Safaee et al. 2012).

The single-stranded helical structure of poly(A) is important in deadenylation, translation quality control, and polyadenylation. The studies described here uncover a new paradigm regarding the recognition of the characteristic structure of single-stranded RNA. The ubiquity of the stacked poly(A) helix in biology suggests that, because the electrostatic distribution and resulting conformation of single-stranded poly(A) is unique among polyribonucleotides, it has been selected as a marker for a correctly processed transcript. This raises the interesting possibility that the sequence of other single-stranded RNAs, such as poly(C), may be indirectly recognized through their conformations.

## Figures and Tables

**Figure 1:**
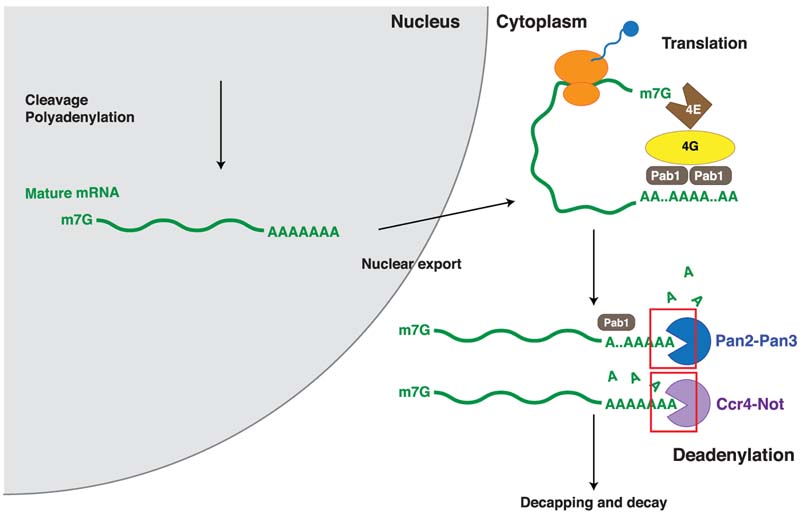
Overview of the role of poly(A) in posttranscriptional regulation of gene expression. The poly(A) tail is added to the 3’ end of nascent transcripts by the cleavage and polyadenylation machinery in the eukaryotic nucleus. The polyadenylated mature mRNA can be exported into the cytoplasm, where it is bound by poly(A)-binding protein to increase the efficiency of translation. Cytoplasmic mRNAs also undergo turnover and decay; the first step of mRNA turnover is the shortening of the poly(A) tail, a process known as deadenylation. Once the poly(A) tail is shortened, translation is inhibited and the transcript undergoes decapping and degradation.
